# Supplementary material for: High fidelity DNA ligation prevents single base insertions in the yeast genome
Source: Nat Commun. 2024 Oct 9;15:8730. doi: 10.1038/s41467-024-53063-1 (PMC11461686; doi:10.1038/s41467-024-53063-1)
Supplement: Supplementary file 1 — Supplementary Information [file 41467_2024_53063_MOESM1_ESM.pdf]

Supplementary Information

High fidelity DNA ligation prevents single base insertions in the yeast genome

Jessica S. Williams, Scott. A. Lujan, Mercedes E. Arana, Adam B. Burkholder, Percy P. Tumbale, R. Scott Williams and Thomas A. Kunkel

a

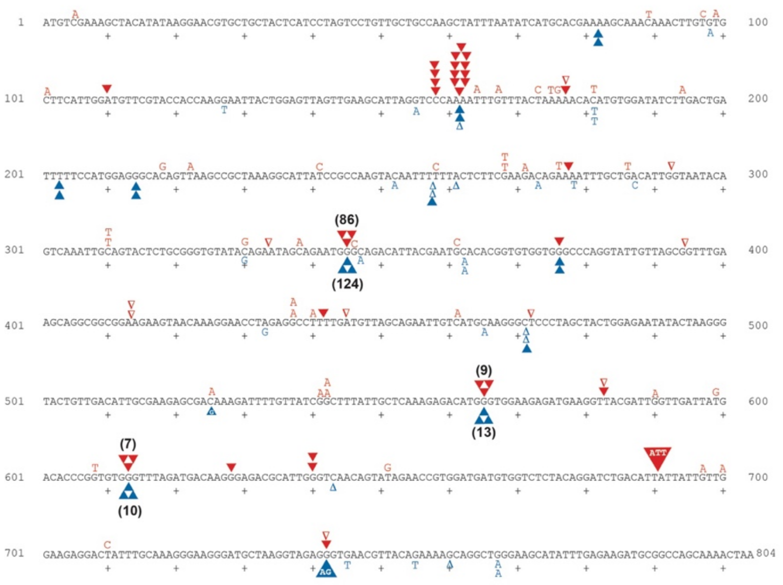

b

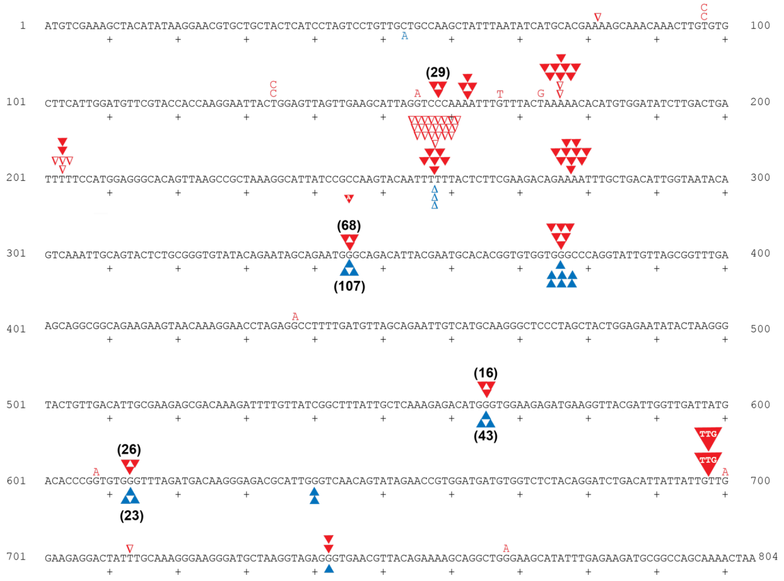

Supplementary Fig. 1 | The *cdc9-EEAA URA3-OR1* and *-OR2* mutation spectra are dominated by +1 frameshifts. The coding strand of the 804 bp *URA3* gene is shown. Small sequence changes (≤ 3 bp) observed in independent *ura3* mutants are depicted above the coding sequence for the *URA3*-orientation 1 (OR1) reporter in red (n=189), and below the coding sequence for the *URA3*-orientation 2 (OR2) in blue (n=190) for (**a**) the *cdc9-EEAA* mutant and (**b**) for *the cdc9-EEAA msh2*Δ mutant (OR1; n = 236 and OR2; n = 189). Letters indicate single-base substitutions, closed triangles indicate +1 frameshifts or ≤ 3 bp insertions (sequence indicated) and open triangles indicate -1 frameshifts. At sites where a large number of events were observed, the number of events is indicated in black.

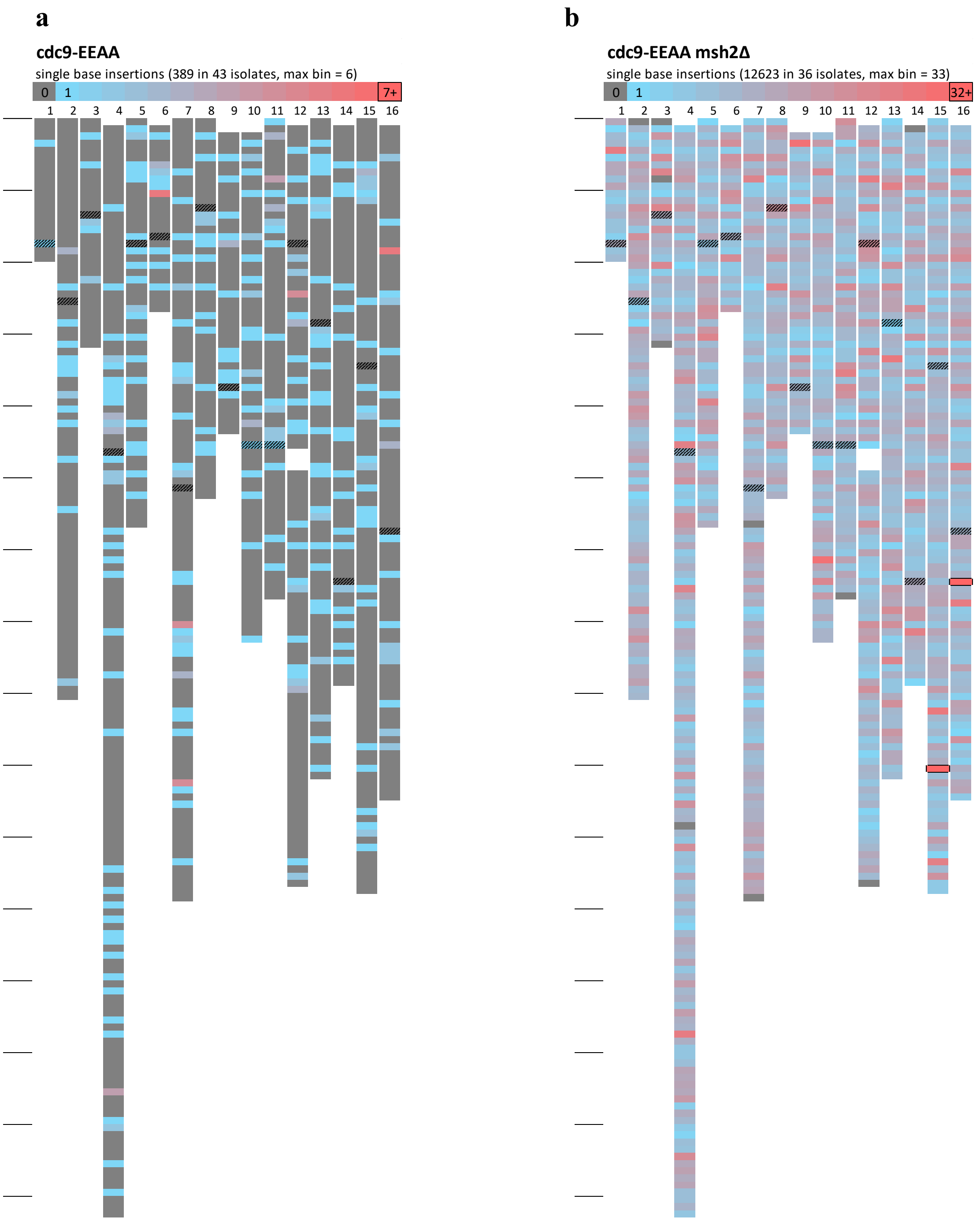

Supplementary Fig. 2 | Single base insertions across the 16 yeast chromosomes. Heatmaps of single-base insertions in 3 bp homopolymers in the *cdc9-EEAA* and *cdc9-EEAA msh2*Δ strains across the 16 *S. cerevisiae* chromosomes (10 kbp bins). Unmapped bins are colored white. All others are colored by the number of observed mutations. As per the key, bins with no mutations are grey, bins with one mutation are blue, bins approaching the significance threshold are red, and intermediate counts transition between blue and red. Bins with counts exceeding the significance threshold have a black border (Šidák correction; each bin counts as a hypothesis tested; family-wise error rate = 0.05). Bins containing centromeres are crosshatched black. (**a**) *cdc9-EEAA*. (**b**) *cdc9-EEAA msh2*Δ. Source data are provided as a Source Data file.

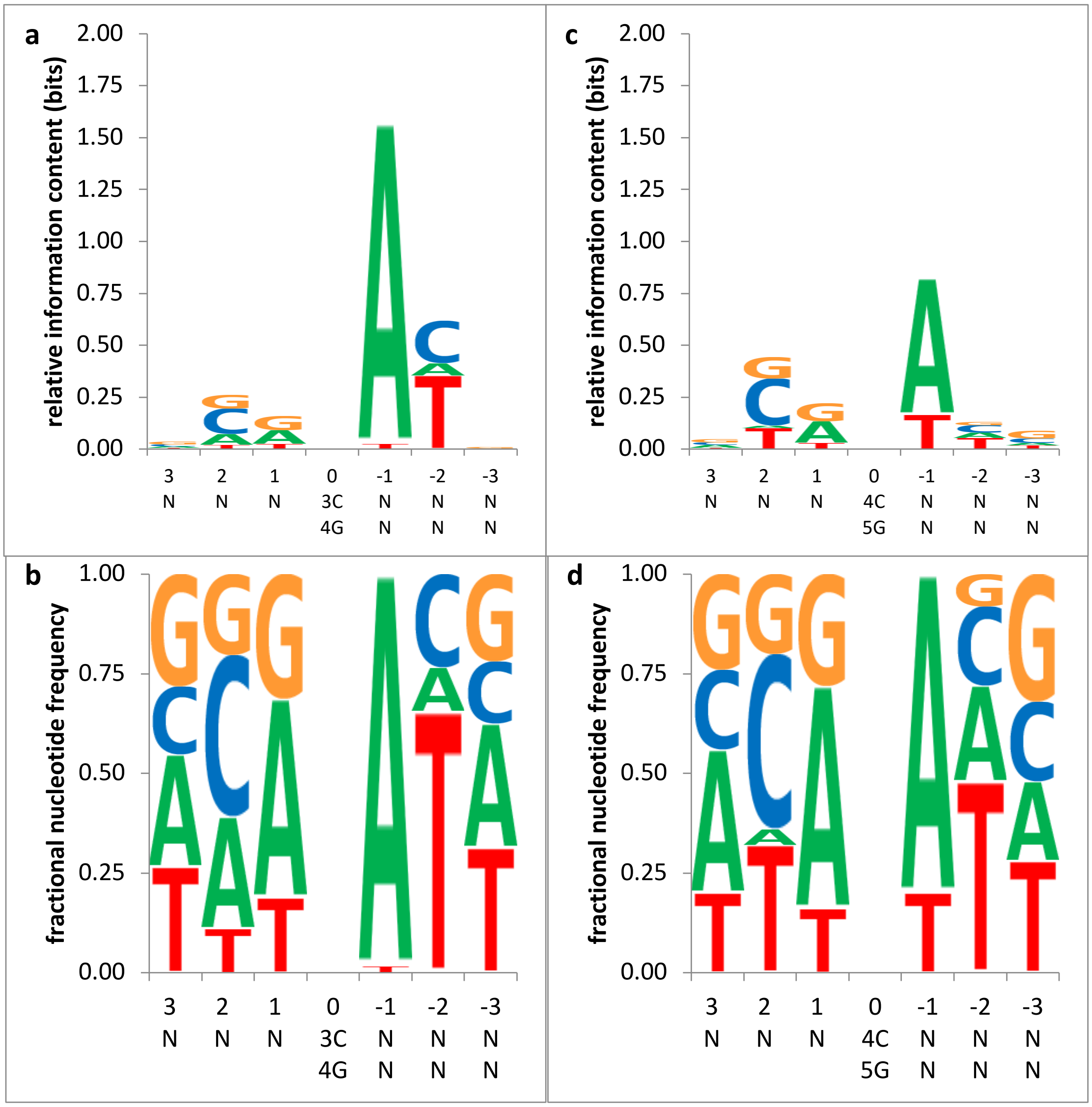

Supplementary Fig. 3 | Sequence context surrounding the +G mutations in the MMR-proficient *cdc9-EEAA* mutant. (**a**) As per Fig. 4e but drawing from only the first and last 50% of each inter-origin space, a sequence logo illustrates the motif for G insertions in the *cdc9-EEAA* strain. Using origin proximity to estimate strandedness, 98% of inferred lagging strand G insertions in 3 bp C-runs (n = 64) are found in runs that are followed by a template A and 89% are then followed by template pyrimidines (C or T). (**b**) Fractional nucleotide frequencies used in the construction of the sequence logo in panel A. (**c**) As per panel a, but for 4 bp C-runs (n = 25). 80% of inferred lagging strand G insertions in these runs are followed by a template A and 68% are then followed by template pyrimidines (C or T). (**d**) As per panel b, but for constructing the sequence logo in panel c. Source data are provided as a Source Data file.

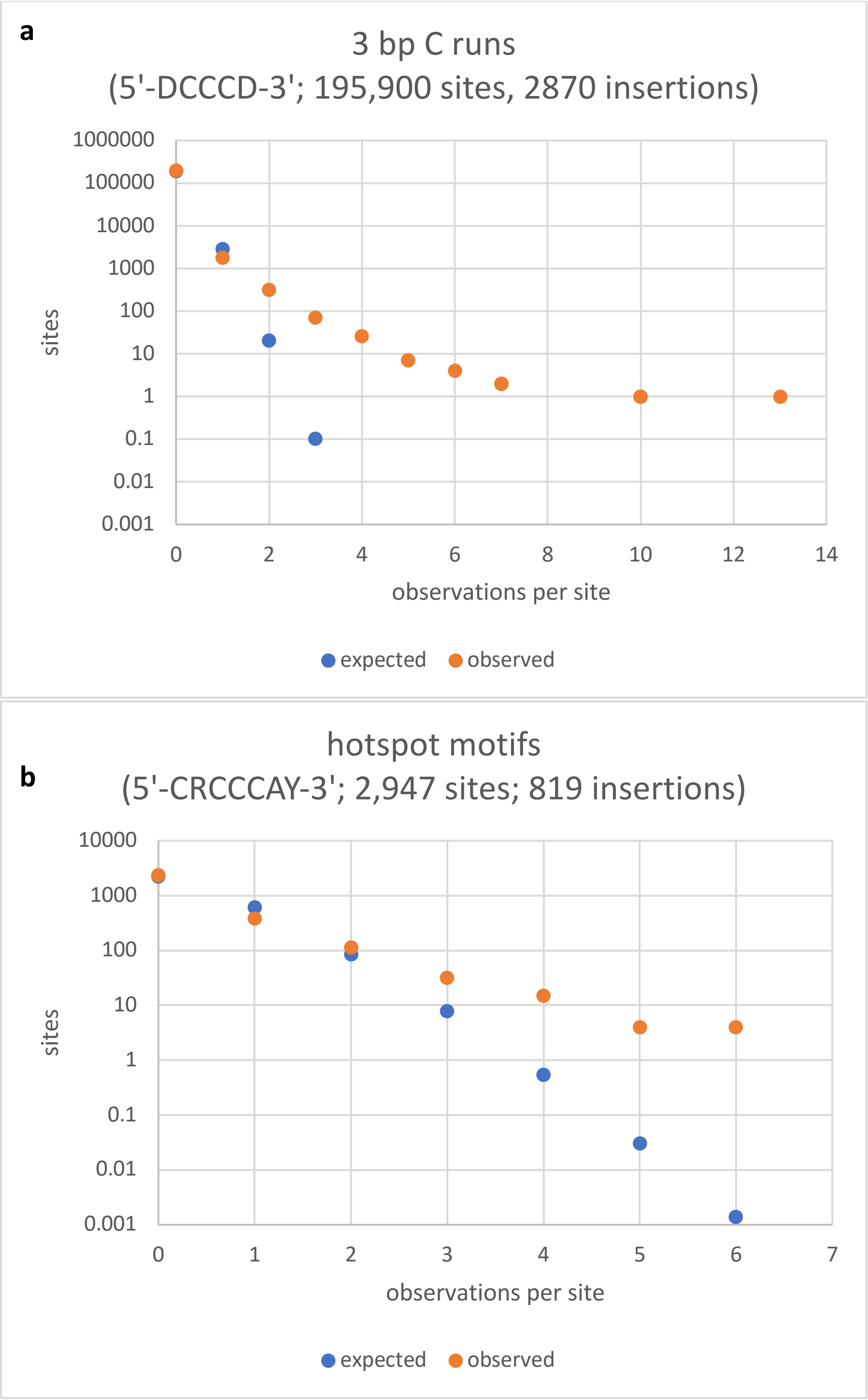

Supplementary Fig. 4 | Mutation Hotspot Statistics. Genomic sites with the indicated sequence were binned by the number of insertion mutations at each site in the *cdc9-EEAA msh2*Δ strain. Observed counts are in orange, predicted counts are in blue. (**a**) Insertion counts for all runs of 3 Cs (5′-DCCCD-3′). (**b**) Insertion counts for all runs of 3 Cs with flank sequences determined from the consensus of the 15 sites with >4 observations (i.e. the hotspot motif; 5′-CRCCCAY-3′). Source data are provided as a Source Data file.

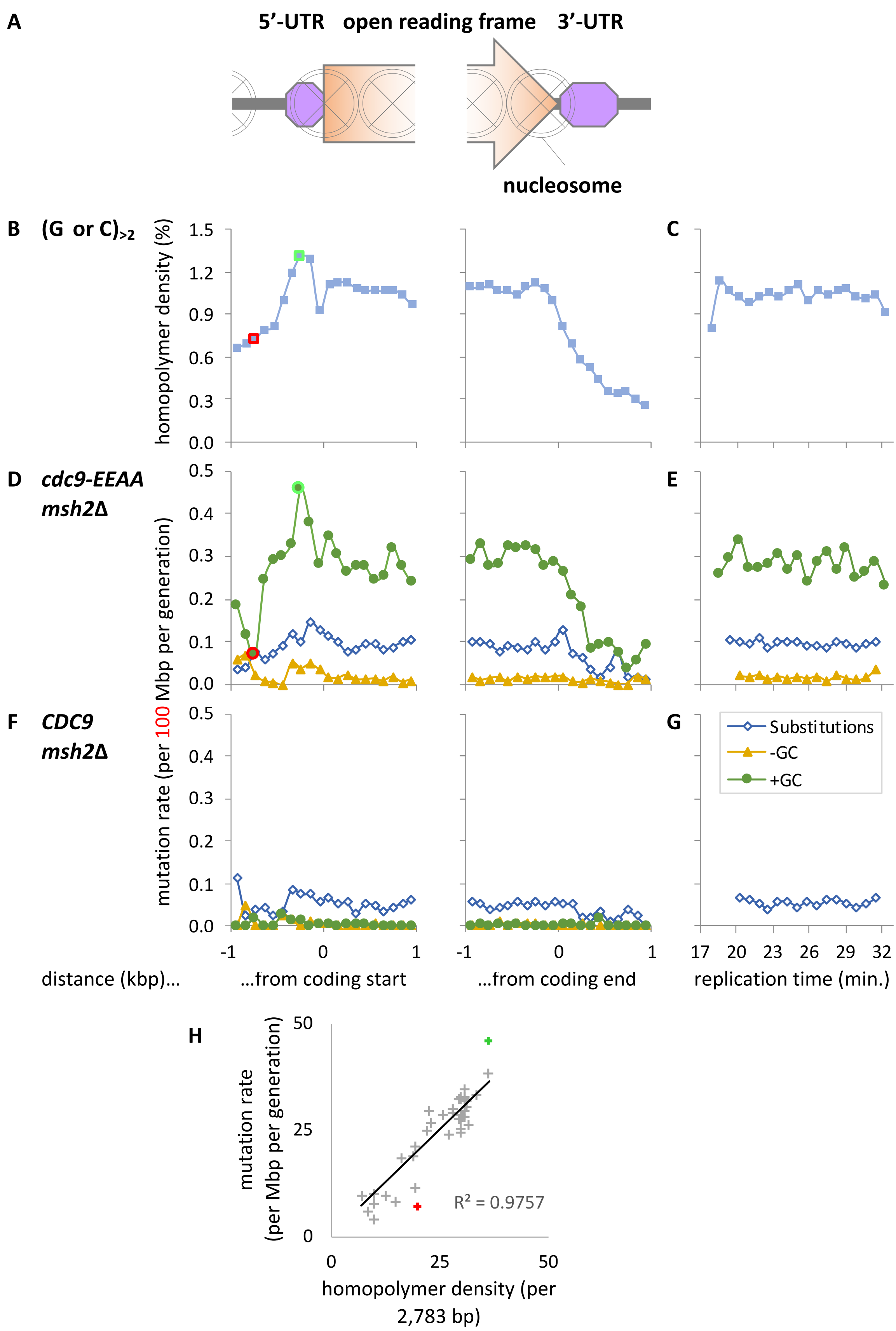
A
5′-UTR
open reading frame
3′-UTR
nucleosome
B
(G or C)>2
homopolymer density (%)
C
D
cdc9-EEAA
msh2Δ
E
F
CDC9
msh2Δ
G
mutation rate (per 100 Mbp per generation)
Substitutions
-GC
+GC
distance (kbp)…
…from coding start
…from coding end
replication time (min.)
H
mutation rate
(per Mbp per generation)
R² = 0.9757
homopolymer density (per
2,783 bp)

Supplementary Fig. 5 | +G mutation rate versus gene positions relative to gene positions, replication timing, and homopolymer density. (**a**) A diagram of protein-coding genes and their surroundings. Features are indicated by shape/color: orange arrow = coding region; purple octagons = untranslated regions (UTRs); and grey circles = average nucleosome positions. Maps around protein-coding genes are bifurcated to account for genes of varying lengths. (**b-c**) The density of G or C homopolymers longer than 2 bp. (**b**) Density around protein coding genes. Red and green-bordered points correspond to the locations of the most extreme insertion rates (see panel **d**). (**c**) Density across replication time, as measured after release from alpha factor arrest [1]. (**d-g**) Data point color and shape denote mutation type: open blue diamonds = substitutions; closed yellow triangles = deletions of G or C; and closed green circles = insertions of G or C. (**d-e**) Mutation rates in the *cdc9-EEAA msh2*Δ strain. (**d**) Rates around protein coding genes. The point of maximum G or C insertions is bordered in green, the minimum in red. (**e**) Rates over replication time. (**f-g**) As per panels **d-e**, but for the *cdc9-EEAA* strain. (**h**) Linear regression (black line) indicates the correlation ($R^2 = 0.976$) between G or C insertion rates (panel **d**) and G or C homopolymer density (panel **b**). Red and green-bordered points correspond to the locations of the most extreme insertion rates (see panel **d**). Source data are provided as a Source Data file.

Supplementary Tables

Supplementary Table 1a. Haploid *S. cerevisiae* strains

Strain	Name(s)	Relevant Genotype	Source
wt	SNM8	*agp1::URA3-OR1*	[2]
wt	SNM18	*agp1::URA3-OR2*	[2]
cdc9-EEAA	YJW1190,91,92,94	*cdc9-E206A-E443A-5FLAG:hphMX6* *agp1::URA3-OR1*	[3]
cdc9-EEAA	YJW1443,6,9,50,51	*cdc9-E206A-E443A-5FLAG:hphMX6* *agp1::URA3-OR2*	This study
msh2∆	YJW1506,7,14,15	*msh2::LEU2*	[3]
cdc9-EEAA *msh2∆*	YJW1500,04,17,18	*cdc9-E206A-E443A-5FLAG:hphMX6* *msh2::LEU2*	[3]

Supplementary Table 1b. Diploid *S. cerevisiae* strains

Strain	Name(s)	Relevant Genotype	Source
wt	yTAKd_005	*agp1::URA3-OR1/agp1::URA3-OR1*	[4]
msh2∆/ *msh2∆*	d0111	msh2::hphMX4/msh2::hphMX4 *agp1::URA3-OR1/agp1::URA3-OR1*	[4]
cdc9-EEAA/ *EEAA*	946-948, 951-953	*cdc9-E206A-E443A-5FLAG:hphMX6/* *cdc9-E206A-E443A-5FLAG:hphMX6* *agp1::URA3-OR1/agp1::URA3-OR1*	This study

Supplementary Table 2. Total and specific mutation counts for the *cdc9-EEAA* +/- *MSH2* strains for the URA3 reporter in OR1 versus OR2

Strain	***wt***		***msh2Δ***		***cdc9-EEAA***		***cdc9-EEAA msh2Δ***	
URA3 orientation	**OR1**	**OR2**	**OR1**	**OR2**	**OR1**	**OR2**	**OR1**	**OR2**
Total	191	239	180	181	189	190	236	189
+1 frameshifts	0	3	10	15	128	160	177	183
+C•G at 344	0	1	0	0	87	124	68	107
+C•G at 564	0	0	0	0	9	13	16	43
+C•G at 612	0	0	0	3	7	10	26	23

The sequencing data for wt and *msh2Δ* is from [2]. The *URA3-OR1* mutation rate and sequencing data for *cdc9-EEAA* +/- *MSH2* strains is from [3].

Supplementary Table 3. Total and specific mutation rates for the *cdc9-EEAA* +/- *MSH2* strains for the URA3 reporter in OR1 versus OR2

Strain	***wt***		***msh2Δ***		***cdc9-EEAA***		***cdc9-EEAA msh2Δ***	
URA3 orientation	**OR1**	**OR2**	**OR1**	**OR2**	**OR1**	**OR2**	**OR1**	**OR2**
Total (x 10^{-8})	1.5	1.5	72	53	4.2	2.3	300	590
+1 frameshifts (x 10^{-8})	≤0.008	0.019	4.0	4.4	2.8	1.9	220	570
+C•G at 344 (x 10^{-8})	≤0.008	0.0063	≤0.4	≤0.29	1.9	1.5	86	330
+C•G at 564 (x 10^{-8})	≤0.008	≤0.0063	≤0.4	≤0.29	0.2	0.16	20	134
+C•G at 612 (x 10^{-8})	≤0.008	≤0.0063	≤0.4	0.88	0.15	0.12	33	72

The sequencing data for wt and *msh2Δ* is from [2]. The *URA3-OR1* mutation rate and sequencing data for *cdc9-EEAA* +/- *MSH2* strains is from [3].

Supplementary Table 4. Insertion bias and correlation

Strain	Context	G/C bias	R^2
cdc9-EEAA	(G or C)$_n$	2.2x	0.422
cdc9-EEAA msh2Δ	(G or C)$_3$	19x	0.969
cdc9-EEAA msh2Δ	(G or C)$_4$	4.4x	0.956
cdc9-EEAA msh2Δ	(G or C)$_5$	5.6x	0.748
Strain	Context	T/A bias	R^2
cdc9-EEAA	(T or A)$_n$	3.9x	0.777
cdc9-EEAA msh2Δ	(T or A)$_4$	8.3x	0.948
cdc9-EEAA msh2Δ	(T or A)$_5$	8.7x	0.881
cdc9-EEAA msh2Δ	(T or A)$_6$	1.3x	0.045

G versus C insertion and T versus A biases from insertion fractions between adjacent origins (Fig. 4a-d and Fig. 5a-d, respectively). Bias at origins (G/C bias and T/A bias) and correlation between inter-origin position and insertion fraction (R^2) were calculated from linear regression of the insertion fraction curves (see Supplementary Data File 1). Source data are provided as a Source Data file.

Supplementary Table 5. Frequency of bases from the *cdc9-EEAA msh2*Δ insertion motifs flanking short homopolymer runs

all C insertions

$p < 10^{-150}$	-2 C	-1 R	CCC	+1 A	+2 Y
N (insert count)	1010	2333	2857	2802	2654
$f_{expected}$	0.198	0.624		0.473	0.527
$f_{observed}$	0.354	0.817		0.981	0.929
$p = 2.3x10^{-21}$	-2 C	-1 R	CCCC	+1 A	+2 Y
N	330	1079	1529	1005	888
$f_{expected}$	0.194	0.617		0.506	0.531
$f_{observed}$	0.216	0.706		0.657	0.581
$p = 1.0x10^{-4}$	-2 C	-1 R	CCCCC	+1 A	+2 Y
N	177	417	646	386	376
$f_{expected}$	0.231	0.637		0.509	0.514
$f_{observed}$	0.274	0.646		0.598	0.582
$p = 2.2x10^{-2}$	-2 C	-1 R	CCCCCC	+1 A	+2 Y
N	44	96	184	94	102
$f_{expected}$	0.325	0.578		0.554	0.458
$f_{observed}$	0.239	0.522		0.511	0.554

C insertions in hotspots

$p < 10^{-150}$	-2 C	-1 R	CCC	+1 A	+2 Y
N	463	955	1047	1045	1012
$f_{expected}$	0.198	0.624		0.473	0.527
$f_{observed}$	0.442	0.912		0.998	0.967
$p = 7.0x10^{-9}$	-2 C	-1 R	CCCC	+1 A	+2 Y
N	97	317	453	310	280
$f_{expected}$	0.194	0.617		0.506	0.531
$f_{observed}$	0.214	0.700		0.684	0.618
$p = 3.9x10^{-5}$	-2 C	-1 R	CCCCC	+1 A	+2 Y
N	81	165	259	164	165
$f_{expected}$	0.231	0.637		0.509	0.514
$f_{observed}$	0.313	0.637		0.633	0.637

all A insertions

$p = 6.1x10^{-81}$		-2 T	-1 C	AAAA	+1 Y
N		412	738	1172	1028
$f_{expected}$		0.299	0.338		0.684
$f_{observed}$		0.352	0.630		0.877
$p = 3.4x10^{-16}$		-2 T	-1 C	AAAAA	+1 Y
N		143	224	401	241
$f_{expected}$		0.302	0.329		0.676
$f_{observed}$		0.357	0.559		0.601

p = 1.8x10^{-4}	-2 T	-1 C	AAAAAA	+1 Y
N	98	148	395	321
$f_{expected}$	0.303	0.327		0.679
$f_{observed}$	0.248	0.375		0.813

A insertions in hotspots

p = 1.3x10^{-23}	-2 T	-1 C	AAAA	+1 Y
N	57	99	120	109
$f_{expected}$	0.299	0.338		0.684
$f_{observed}$	0.475	0.825		0.908
p = 1.5x10^{-4}	-2 T	-1 C	AAAAA	+1 Y
N	18	27	40	25
$f_{expected}$	0.302	0.329		0.676
$f_{observed}$	0.450	0.675		0.625

The significance of motifs flanking homopolymers with insertions in the *cdc9-EEAA msh2*Δ strain. Base identities and positions (e.g. '-2 C') are all given relative to the homopolymer oriented 5′-to-3′ in the lagging template strand. N denotes the number of insertions observed with the base identity. The count under the homopolymer (e.g. 'CCC') is the total number of insertions observed in homopolymers of that type and length (N_{total}). Expected fractions ($f_{expected}$) were calculated from the flanks of all homopolymers of the given type and length in the 26% of the genome where the leading/lagging strand template is known for >90% of replications from ribonucleotide mapping [5]. The p-values are from the Chi-squared tests of observed stated versus expected counts (N_{total} x $f_{expected}$).

Supplementary References

1. Muller, C.A. & Nieduszynski, C.A. Conservation of replication timing reveals global and local regulation of replication origin activity. *Genome Res* **22**, 1953-62 (2012).
2. Larrea, A.A. et al. Genome-wide model for the normal eukaryotic DNA replication fork. *Proc Natl Acad Sci U S A* **107**, 17674-9 (2010).
3. Williams, J.S. et al. High-fidelity DNA ligation enforces accurate Okazaki fragment maturation during DNA replication. *Nat Commun* **12**, 482 (2021).
4. Lujan, S.A. et al. Heterogeneous polymerase fidelity and mismatch repair bias genome variation and composition. *Genome Res* **24**, 1751-64 (2014).
5. Zhou, Z.X., Lujan, S.A., Burkholder, A.B., Garbacz, M.A. & Kunkel, T.A. Roles for DNA polymerase delta in initiating and terminating leading strand DNA replication. *Nat Commun* **10**, 3992 (2019).
