# Supplementary material for: High fidelity DNA ligation prevents single base insertions in the yeast genome
Source: Nat Commun. 2024 Oct 9;15:8730. doi: 10.1038/s41467-024-53063-1 (PMC11461686; doi:10.1038/s41467-024-53063-1)
Supplement: Supplementary file 3 — Description of Additional Supplementary Files [file 41467_2024_53063_MOESM3_ESM.pdf]

Description of Additional Supplementary Files

File Name: Supplementary Data 1
Description: Genomic mutation counts and rates for all mutation types
